# Expert perspectives on ECCO_2_R for acute hypoxemic respiratory failure: consensus of a 2022 European roundtable meeting

**DOI:** 10.1186/s13613-024-01353-8

**Published:** 2024-08-22

**Authors:** Alain Combes, Georg Auzinger, Luigi Camporota, Gilles Capellier, Guglielmo Consales, Antonio Gomis Couto, Wojciech Dabrowski, Roger Davies, Oktay Demirkiran, Carolina Ferrer Gómez, Jutta Franz, Matthias Peter Hilty, David Pestaña, Nikoletta Rovina, Redmond Tully, Franco Turani, Joerg Kurz, Kai Harenski

**Affiliations:** 1 Institute of Cardiometabolism and Nutrition, INSERM Unité Mixte de Recherche (UMRS) 1166, Sorbonne Université, 47, Boulevard de l’Hôpital, 75013 Paris, France; 2 Service de Médecine Intensive-Réanimation, Sorbonne Université, Hôpital Pitié-Salpêtrière, Assistance Publique-Hôpitaux de Paris, 75013 Paris, France; 3 Department of Critical Care, King’s College Hospital, London, SE5 9RS UK; 4 Department of Critical Care, Cleveland Clinic, London, SW1Y 7SW UK; 5 Department of Critical Care, Guy’s & St Thomas’ NHS Foundation Trust, London, SE1 7EH UK; 6 Centre for Human and Applied Physiological Sciences, School of Basic and Medical Biosciences, King’s College London, London, SE1 1UL UK; 7 University of Franche-Comté, 25000 Besançon, France; 8 Department of Epidemiology and Health, Monash University, Melbourne, VIC 3004 Australia; 9 Anesthesia, Intensive Care and Emergency Department, Prato Hospital, Azienda Toscana Centro, Prato, Italy; 10 Servicio de Nefrología, Hospital Universitario Ramón y Cajal, 28033 Madrid, Spain; 11 First Department of Anaesthesiology and Intensive Therapy, Medical University of Lublin, 20-954 Lublin, Poland; 12 Chelsea and Westminster Hospital NHS Foundation Trust, London, SW10 9NH UK; 13 Division of Anaesthetics, Intensive Care and Pain Medicine, Imperial College London, Chelsea and Westminster Hospital Campus, London, SW10 9NH UK; 14 Department of Anesthesiology and Intensive Care, Cerrahpaşa Medical Faculty, Istanbul University-Cerrahpaşa, Istanbul, 34098 Turkey; 15 Anesthesiology and Intensive Care Department, Consorcio Hospital General Universitario de Valencia, 46014 Valencia, Spain; 16 Department of Cardiology and Internal Intensive Care, Rems-Murr-Kliniken Winnenden, 71364 Winnenden, Germany; 17 Institute of Intensive Care Medicine, University Hospital Zurich, 8091 Zurich, Switzerland; 18 Servicio de Anestesia-Reanimación, Hospital Universitario Ramón y Cajal, Carretera de Colmenar Km 9, 28034 Madrid, Spain; 19 Facultad de Medicina, Instituto Ramón y Cajal de Investigación Sanitaria, Universidad de Alcalá, 28034 Madrid, Spain; 20 1st Respiratory Department, National and Kapodistrian University of Athens Medical School, “Sotiria” Chest Hospital, 152 Mesogion Av, 11527 Athens, Greece; 21 Royal Oldham Hospital, Northern Care Alliance NHS Trust, Oldham, OL1 2JH UK; 22 Department of Intensive Care, Aurelia Hospital, Via Aurelia 860, 00165 Rome, Italy; 23 Cardiac Anaesthesia European Hospital, Via Portuense, 760, 00416 Rome, Italy; 24 Baxter Healthcare, Edisonstr 4, 85716 Unterschleißheim, Germany

**Keywords:** Acidosis, ECCO_2_R, Hypercapnia, Lung protective ventilation, Mechanical ventilation, Ultra-protective lung ventilation

## Abstract

**Background:**

By controlling hypercapnia, respiratory acidosis, and associated consequences, extracorporeal CO_2_ removal (ECCO_2_R) has the potential to facilitate ultra-protective lung ventilation (UPLV) strategies and to decrease injury from mechanical ventilation. We convened a meeting of European intensivists and nephrologists and used a modified Delphi process to provide updated insights into the role of ECCO_2_R in acute respiratory distress syndrome (ARDS) and to identify recommendations for a future randomized controlled trial.

**Results:**

The group agreed that lung protective ventilation and UPLV should have distinct definitions, with UPLV primarily defined by a tidal volume (V_T_) of 4–6 mL/kg predicted body weight with a driving pressure (ΔP) ≤ 14–15 cmH_2_O. Fourteen (93%) participants agreed that ECCO_2_R would be needed in the majority of patients to implement UPLV. Furthermore, 10 participants (*majority*, 63%) would select patients with PaO_2_:FiO_2_ > 100 mmHg (> 13.3 kPa) and 14 (*consensus*, 88%) would select patients with a ventilatory ratio of > 2.5–3. A minimum CO_2_ removal rate of 80 mL/min delivered by continuous renal support machines was suggested (11/14 participants, 79%) for this objective, using a short, double-lumen catheter inserted into the right internal jugular vein as the preferred vascular access. Of the participants, 14/15 (93%, *consensus*) stated that a new randomized trial of ECCO_2_R is needed in patients with ARDS. A ΔP of ≥ 14–15 cmH_2_O was suggested by 12/14 participants (86%) as the primary inclusion criterion.

**Conclusions:**

ECCO_2_R may facilitate UPLV with lower volume and pressures provided by the ventilator, while controlling respiratory acidosis. Since recent European Society of Intensive Care Medicine guidelines on ARDS recommended against the use of ECCO_2_R for the treatment of ARDS outside of randomized controlled trials, new trials of ECCO_2_R are urgently needed, with a ΔP of ≥ 14–15 cmH_2_O suggested as the primary inclusion criterion.

**Supplementary Information:**

The online version contains supplementary material available at 10.1186/s13613-024-01353-8.

## Background

Clinical data suggest that mechanical ventilation (MV) can contribute to the negative outcomes in patients with acute respiratory distress syndrome (ARDS) through ventilator-induced lung injury (VILI) [1–3]. The ARDSNet investigators demonstrated that limiting tidal volume (V_T_) to 6 mL/kg of predicted body weight (PBW) and plateau pressure (P_Plat_) to < 30 cm H_2_O improved survival. However, this approach may not be fully protective as ~ 30% of patients exhibit tidal hyperinflation along with an increase in proinflammatory mediators in bronchoalveolar lavage fluid, a typical signal for VILI [4, 5]. Reducing V_T_ even further to 4 mL/kg and P_Plat_ to < 25 cmH_2_O, a strategy termed “ultra-protective ventilation”, has been proposed to reduce VILI effects [6–8]. Furthermore, other variables of risk reduction for VILI have been discussed: Amato et al. [9] suggested reduction of driving pressure (ΔP), reduction of respiratory rate [10, 11] and/or mechanical power [12–15] are other variables to be discussed in this context. However, this strategy entails the risks associated with hypercapnia and severe respiratory acidosis [16–18].

As an adjunct to MV, extracorporeal CO_2_ removal (ECCO_2_R) aims to clear CO_2_, enabling ultra-protective lung ventilation while limiting hypercapnia and respiratory acidosis [19–22]. In 2019, a European ECCO_2_R user group meeting identified factors influencing patient selection and clinical decision-making, as well as how to implement ECCO_2_R in the intensive care unit (ICU) [17]. The group considered ARDS to be the primary indication for ECCO_2_R, with the treatment goal being to facilitate ultra-protective lung ventilation by decreasing V_T_, P_Plat_, ΔP, and RR [17].

Since this framework was proposed, experience of ECCO_2_R and ultra-protective lung ventilation has increased. The COVID-19 pandemic provided experience of delivering ECCO_2_R to different patient groups [23–25]. While the REST study (NCT02654327) reported no difference in 90-day mortality in patients receiving ultra-low V_T_ ventilation (ULTV) with ECCO_2_R compared with those receiving low V_T_ ventilation (LTV) without ECCO_2_R [26], a secondary analysis suggested that the use of ECCO_2_R may improve survival in patients with a ventilatory ratio (VR, ≥ 3; a simple bedside index of impaired efficiency of ventilation, which correlates well with physiological dead space fraction) [27, 28]. However, uncertainty remains around the use of ECCO_2_R in ARDS, and the recent European Society of Intensive Care Medicine (ESICM) guidelines recommended that the use of ECCO_2_R for the treatment of ARDS should be limited to randomized controlled trials [29]. We therefore convened the second European ECCO_2_R Expert Roundtable Meeting to update the framework for ECCO_2_R and to outline further research in this area.

## Methods

### Participants

The ECCO_2_R Expert Roundtable Meeting was held in Brussels on the 5 October 2022 and was attended by 16 clinicians (1 chair [AC] plus 15 respondents) who regularly provide ECCO_2_R in clinical centers across Europe. Each participant was a senior clinician or an intensivist with direct clinical experience of ECCO_2_R, with several of the participants being principal investigators in recently completed or ongoing clinical trials. JK and KH are employees of Baxter who were engaged in the development of the questionnaire. They did not participate in the roundtable discussion but, like all other authors, they participated in drafting the manuscript and critically revising it for important intellectual content. There was no modification of the intellectual content by Baxter employees other than the listed authors. All authors take responsibility for the final content of the manuscript. Conflict of interest declarations for the attendees can be found at the end of the manuscript.

### Objectives

The objectives of the Expert Roundtable Meeting were to understand current clinical practice for ECCO_2_R in patients with acute hypoxemic respiratory failure, including the clinical rationale for the use of ECCO_2_R, the criteria used for initiation, maintenance, and discontinuation in patients with mild-to-moderate ARDS, and practical considerations, including anticoagulation and vascular access strategies. The meeting also aimed to assess the impact of recent publications investigating the use of ECCO_2_R to support ultra-protective lung ventilation for acute respiratory failure [26], as well as the impact of the COVID-19 pandemic on current and future standards of practice.

### Data collection and analysis

A modified Delphi-based methodology was used to assess the clinicians’ views on ECCO_2_R over four rounds of iterative questioning, including an anonymous pre-meeting survey, a live survey during the meeting, and two anonymous post-meeting surveys (Table 1). The meeting questions, as well as the pre-meeting and post-meeting surveys, were developed by AC in collaboration with JK, with independent medical writing support funded by Baxter. The questions are available in the supplementary appendix. JK and KH did not participate in answering the surveys.

**Table 1:** Overview of the modified Delphi method

	Objective(s)	Steps	Format
1. Preparation phase	• Gain a full understanding of the current experience of providing ECCO_2_R in the ICU	• Identify and review relevant literature • Identify and recruit physicians with experience of ECCO_2_R	• Systematic literature search
2. Pre-meeting survey	• Confirm the baseline of ECCO_2_R experience within the team • Inform key topics to be covered in the Roundtable Meeting	• Develop questions based on practice identified in the literature • Blinded analysis of responses	• Anonymous PDF questionnaire shared by email
3. Roundtable Meeting	• Gain a full understanding of the use and practice of ECCO_2_R	• Full day meeting with independent facilitator • Questions presented to attendees, followed by open discussion and blinded voting	• Independently facilitated meeting
4. Post-meeting survey 1	• Refine and clarify open topics identified in the meeting	• Develop questions based on feedback from the meeting • Blinded analysis of responses	• Anonymous PDF questionnaire shared by email
5. Post-meeting survey 2	• Understand the impact of new publications and guidelines on perceptions of ultra-protective ventilation	• Develop questions based on literature published post-meeting • Blinded analysis of responses	• Anonymous PDF questionnaire shared by email
6. Report development	• Disseminate findings	• Manuscript developed in alignment with GPP	• Manuscript

Each step was a distinct process that was completed before the following step was initiated. Results and discussions from each step were independently analyzed and informed the direction and content of the subsequent step, e.g. if the group were split on a topic, then clarifying questions were crafted to guide the discussions in the following step(s) to identify and explore points of consensus or difference

*ECCO*_*2*_*R* extracorporeal carbon dioxide removal, *GPP* Good Publication Practice, *ICU* intensive care unit, *PDF* portable document format

The Round 1 pre-meeting survey consisted of a PDF questionnaire that was circulated to each participant individually in advance of the meeting, with results analyzed anonymously. Results from the Round 1 survey were presented to the group and used to inform the questions asked in the Round 2 meeting, which was moderated by an independent facilitator. In Round 2, participants were divided into four subgroups and questions were presented to the group by an independent facilitator. For closed questions, participants provided their responses anonymously through a web-based voting system. For open questions, responses from each group were collected after a period of discussion to facilitate interaction between participants. JK and KH were present as Baxter employees during the meeting but were not permitted to provide answers or responses. To further explore questions and topics raised during the meeting, a first post-meeting survey (Round 3) was shared with the authors. Based on the Round 3 survey and literature published following the meeting, including the secondary analysis of the REST trial, the ESICM guidelines and the VT4COVID trial [28–30], a second post-meeting survey (Round 4) was shared with the authors to understand their definition of ultra-protective ventilation and the role of ECCO_2_R. Both Round 3 and 4 surveys consisted of PDF questionnaires that were shared with each participant individually and the results were analyzed anonymously.

Responses to the survey questions at each round were evaluated to determine the level of agreement between participants. A threshold of ≥ 80% of participants in agreement was defined as a “*consensus*.” A threshold of ≥ 50% of participants in agreement was defined as a “*majority*,” while < 50% was defined as “*no agreement*.” These thresholds are consistent with the analysis conducted in 2019 [17].

To facilitate the analysis of responses for questions regarding respiratory parameters used for the implementation of lung protective ventilation and ECCO_2_R, a ranked scoring system was employed. Participants were asked to score respiratory parameters in order of perceived importance, giving them a score (e.g. from 1 to 5, depending on the number of variables). Scores were then combined to give a total score for each parameter, with higher scores indicating a higher perceived importance.

## Results

### Participant experience

The participants at the meeting were experienced clinicians who regularly provide ECCO_2_R in clinical centers across Europe; the average number of patients treated with ECCO_2_R therapy in their ICU/unit per year was 8 (range 1–18). Regarding patients with mild-to-moderate ARDS (defined by the 2012 Berlin definition), the average number of admissions per year to their ICU/unit was 112 (range 40–250). Participants used all available devices for ECCO_2_R present in the EU at that time, including devices from ALung, B-Braun and Fresenius. The *majority* of participants (93%) had experience with Baxter’s product PrismaLung +.

### Definitions of lung protective ventilation and ultra-protective lung ventilation

**Fig. 1:**
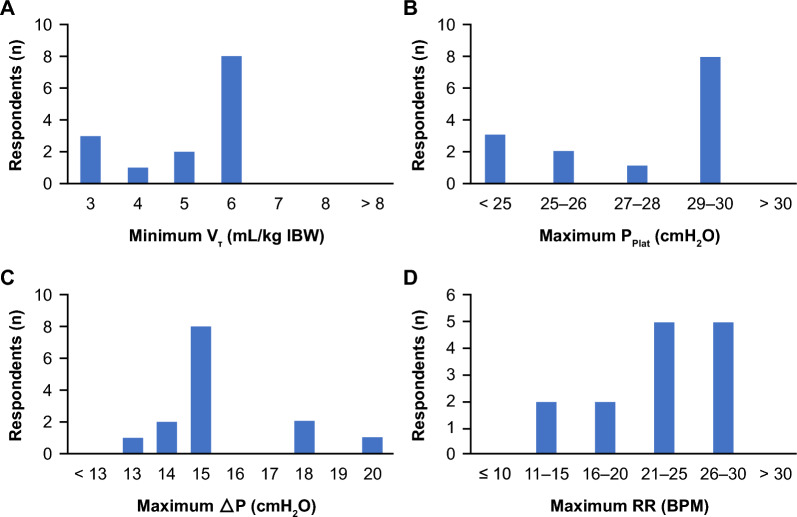
Acceptable threshold values for respiratory parameters when implementing protective ventilation to minimize or avoid VILI for a patient with mild-to-moderate ARDS. 14/15 participants answered this question. ≥ 12 responses indicate *consensus*, ≥ 7 responses indicate a *majority*, and < 7 responses indicate *no agreement*

**Fig. 2:**
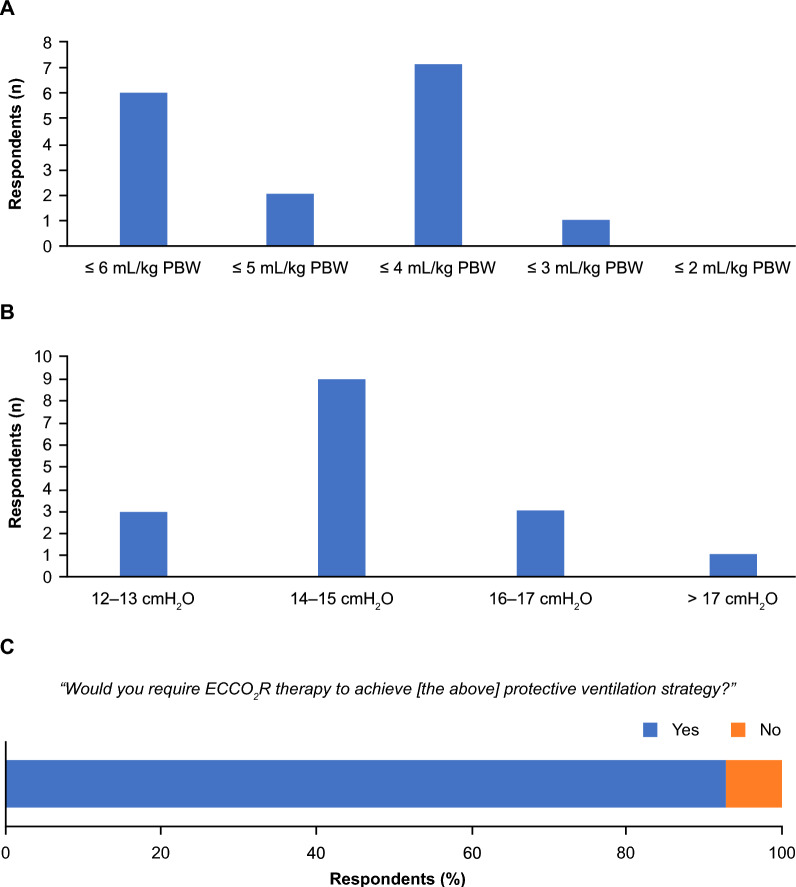
Ventilatory objectives for ultra-protective lung ventilation. Target V_T_ (**A**) and driving pressure (**B**) thresholds for ultra-protective lung ventilation as defined by the participants. (**C**) Rate of participants stating that ECCO_2_R would be required to implement ultra-protective lung ventilation. All participants answered this question. ≥ 12 responses indicate a *consensus*, ≥ 8 responses indicate a *majority*, and ≤ 7 responses indicate *no agreement*

The *majority* of participants (57%) agreed that lung protective ventilation and ultra-protective lung ventilation should have distinct definitions, with lower targets for V_T_, ΔP, P_Plat_, and RR identified as the parameters that define ultra-protective lung ventilation vs. more conventional lung protective ventilation. Participants ranked V_T_, ΔP, P_Plat_, and RR as the four most important respiratory parameters to monitor when implementing a protective ventilation strategy. In subsequent rounds, participants agreed that a protective ventilation strategy for patients with mild-to-moderate ARDS should have a target V_T_ of 6 mL/kg PBW, maximum ΔP of 15 cmH_2_O, and maximum P_Plat_ of 29–30 cmH_2_O (*majority*, Fig. 1A–C). *No agreement* was reached on maximum RR, with 10 participants selecting values of 21–30 breaths per minute (BPM) (Fig. 1D). Based on the ECCO_2_R data published after the meeting, the *majority* of participants (63%) indicated that ultra-protective lung ventilation should have a maximum V_T_ ≤ 6 mL/kg PBW, with 6, 2, 7, and 1 participants defining V_T_ as ≤ 6, ≤ 5, ≤ 4, and ≤ 3 mL/kg PBW, respectively (Fig. 2A). The *majority* of participants consider a VT < 4 mL/kg as the definition for ULTV.

### Use of ECCO_2_R to facilitate protective ventilation in patients with ARDS

All participants indicated that ultra-protective lung ventilation facilitated by ECCO_2_R would require a maximum ΔP (100%, *consensus*), with the *majority* (56%) selecting 14–15 cmH_2_O as their preference; 12–13 cmH_2_O and 16–17 cmH_2_O were selected by three participants each (19%, Fig. 2B). Furthermore, 10 participants (*majority*, 63%) would select patients using a minimum PaO_2_:FiO_2_ of > 100 mmHg (> 13.3 kPa) and 14 (*consensus*, 88%) would select a minimum VR of > 2.5–3. Fourteen (93%) participants agreed that ECCO_2_R would be needed in the majority of patients to implement ultra-protective lung ventilation (*consensus*) (Fig. 2C).

### Initiation and discontinuation of ECCO_2_R

Partial pressure of CO_2_ (PaCO_2_), pH, ΔP, and RR were ranked as the four most important respiratory parameters to consider when deciding whether to initiate ECCO_2_R in a patient who is sedated and ventilated with mild-to-moderate ARDS. In subsequent rounds, the majority agreed they would initiate ECCO_2_R once PaCO_2_ reached > 60 mmHg (> 8 kPa) and pH < 7.25 (both *majority*) (Table 2). *No agreement* was reached on ΔP threshold; however, 10 participants selected either > 14 or > 15 cmH_2_O. *No agreement* was reached on a threshold for RR.

**Table 2:** Initiation and discontinuation thresholds for respiratory parameters when implementing ECCO_2_R in a sedated patient with mild-to-moderate ARDS

Criteria for initiation	Threshold value	Level of agreement
pH	< 7.25	7/13, majority^b^
PaCO_2_	> 60 mmHg	8/14, majority^c^
ΔP	–	No agreement
RR	–	No agreement
Criteria for discontinuation^a^		
pH	> 7.3	7/14, majority^c^
ΔP	–	No agreement
RR	–	No agreement
P_plat_	–	No agreement

^a^These criteria should be evaluated with sweep gas off on the ECCO_2_R device

^b^Two participants declined to answer this question; level of agreement has been calculated using the total number of respondents

^c^One participant declined to answer this question; level of agreement has been calculated using the total number of respondents

A threshold of ≥ 80% of participants in agreement was defined as “*consensus*.” A threshold of ≥ 50% of participants in agreement was defined as a “*majority*,” while < 50% was defined as “*no agreement*.”

*ΔP* driving pressure, *ARDS* acute respiratory distress syndrome, *ECCO*_*2*_*R* extracorporeal carbon dioxide removal, *PaCO*_*2*_ partial pressure of carbon dioxide, *P*_*plat*_ plateau pressure, *RR* respiratory rate

When discontinuing ECCO_2_R, pH, ΔP, RR, and P_plat_ were indicated as being the four most important respiratory parameters to consider (evaluated with sweep gas off on the ECCO_2_R device). In subsequent rounds, most participants would discontinue ECCO_2_R once pH reached > 7.3 (*majority*) (Table 2). *No agreement* was reached on thresholds for ΔP, RR, or P_plat_.

### Anticoagulation strategy for ECCO_2_R

Fourteen out of 15 participants (93%) would use unfractionated heparin for anticoagulation when implementing ECCO_2_R (*consensus*), with one participant stating they would use regional citrate-based anticoagulation (although this treatment is not recommended with flow higher than 150 mL/min) (Table 3). Among participants who preferred heparin, the majority agreed they would target an activated partial thromboplastin time ratio of 1.5–2.0 × control (*majority*). Half of participants (7/14) reported using anti-Xa testing, with all of them agreeing on a target range of 0.3–0.5 IU/mL (*consensus*). *No agreement* was reached on bolus or infusion dosage for unfractionated heparin.

**Table 3:** Anticoagulation strategy when implementing ECCO_2_R in patients who are sedated and ventilated

Preferred anticoagulant^a^	Participants
Unfractionated heparin	14
Citrate	1

Heparin protocol	Range	Level of agreement
aPTT target (ratio vs. reference)	1.5–2.0	9/14, majority^b^
Anti-Xa target (units/mL)	0.3–0.5	7/7, consensus^c^
Bolus dose (units/kg)	–	No agreement
Infusion dose (units/kg/hour)	–	No agreement

^a^All participants answered this question

^b^All participants who used heparin (14/15) answered this question

^c^Level of agreement for this question was calculated using the total number of respondents who use anti-Xa monitoring

A threshold of ≥ 80% of participants in agreement was defined as “*consensus*.” A threshold of ≥ 50% of participants in agreement was defined as a “*majority*,” while < 50% was defined as “*no agreement*”

### Optimal blood flow rate for ECCO_2_R and vascular access

**Fig. 3:**
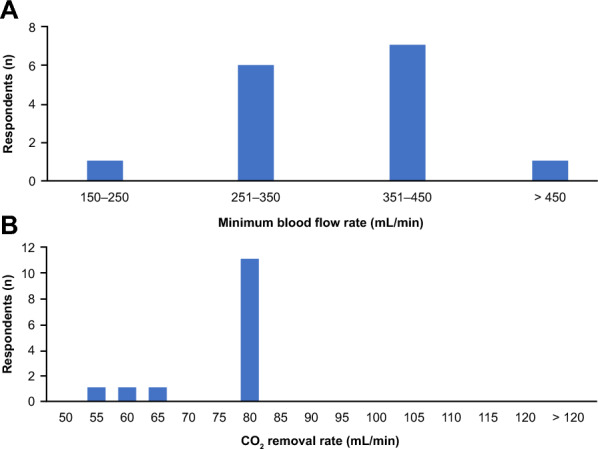
Minimum requirements for ECCO_2_R therapy. **A** Minimum blood flow rate required for effective use of ECCO_2_R. All 15 participants answered this question. ≥ 12 responses indicate *consensus*, ≥ 8 responses indicate a *majority*, and ≤ 7 responses indicate *no agreement*. **B** Minimum CO_2_ removal rate for an ECCO_2_R device. 14/15 participants answered this question. ≥ 12 responses indicate *consensus*, ≥ 7 responses indicate a *majority*, and < 7 responses indicate *no agreement*

Most participants selected either a range of 251–350 mL/min or 351–450 mL/min as the minimum blood flow rate they believed was required for effective use of ECCO_2_R (Fig. 3A). A *majority* of participants (73%) believed that a minimum CO_2_ removal rate of 80 mL/min delivered by technology based on peristaltic (roller) pumps as in renal support devices was required for ECCO_2_R to be effective (Fig. 3B). The right internal jugular vein was the preferred vascular access point for the *majority* of participants (Table 4); however, femoral access was discussed as suitable, especially in conscious patients. All participants preferred using a double-lumen catheter (*consensus*), with most participants specifying a length of 16–17 cm (for the right internal jugular access) and a diameter of 14 French (both *majority*). All considered the use of vascular ultrasound necessary to safely guide venous catheter insertion using the Seldinger technique (*consensus*) (Table 5).

**Table 4:** Vascular access strategy when implementing ECCO_2_R

Parameter	Preference	Level of agreement
Access point	Right internal jugular vein	15/15, consensus
Catheter type	Double-lumen	15/15, consensus
Catheter length	16–17 cm	8/13, majority^a^
Catheter size	14 French	9/13, majority^a^
Vascular assessment	Ultrasound/sonography	15/15, consensus

^a^Two participants declined to answer this question; level of agreement has been calculated using the total number of respondents

A threshold of ≥ 80% of participants in agreement was defined as “*consensus*.” A threshold of ≥ 50% of participants in agreement was defined as a “*majority*,” while < 50% was defined as “*no agreement*”

**Table 5:** Preliminary suggestions for the design of a future randomized trial of ECCO_2_R for patients with acute hypoxemic respiratory failure

Criteria	Threshold value	Level of agreement
Inclusion criteria		
ΔP	≥ 14 or 15 cm H_2_O	Consensus
Minimum PaO_2_:FiO_2_	50–100	No agreement
Maximum PaO_2_:FiO_2_	150–300	No agreement
Minimum PEEP	5–15	No agreement
pH	< 7.20–7.25	No agreement
PaCO_2_	> 60 mmHg	No agreement
RR	> 25	No agreement
Mechanical power	–	No agreement
Exclusion criteria		
Contraindication to heparin	–	No agreement
High risk of bleeding	–	No agreement
Hemodynamic instability	–	No agreement
Major comorbidity	–	No agreement
Primary endpoint		
Mortality		No agreement
Time on invasive ventilation		No agreement
Improvement of physiological parameters (PaO_2_, ΔP, mechanical power)		No agreement
Secondary endpoints		
Time on invasive ventilation		No agreement
Mortality		No agreement
Improvement in right ventricular function		No agreement
Safety endpoints		
Major bleeding (including CNS hemorrhage)		Majority
Catheter-associated complication (infection, vascular injury)		No agreement
Hemolysis		No agreement

ΔP driving pressure, CNS central nervous system, ECCO_2_R extracorporeal carbon dioxide removal, FiO_2_ fraction of inspired oxygen, PaCO_2_ partial pressure of carbon dioxide, PEEP positive end-expiratory pressure, pH potential of hydrogen, RR respiratory rate

### Use of neuromuscular blocking agents and prone positioning during ECCO_2_R

Most participants (9/14, 64%, *majority*) use neuromuscular blockade in patients who are sedated with ventilator asynchrony receiving ECCO_2_R. They (8/13, 62%, *majority)* reported routinely using prone positioning for sedated and ventilated patients with ARDS who are receiving ECCO_2_R.

### Need for and design of another randomized trial of ECCO_2_R for patients with acute hypoxemic respiratory failure

During the post-meeting survey, 14/15 (93%, *consensus*) participants stated that a new randomized trial of ECCO_2_R is needed in patients with ARDS. A ΔP of ≥ 14–15 cmH_2_O was suggested by 12/14 participants (86%) as the primary inclusion criterion. *No agreement* existed for the primary endpoint, although mortality and the duration of MV were mentioned as suitable outcome parameters. Major bleeding (including central nervous system hemorrhage) was the most frequently indicated safety endpoint (8/12, 67%).

## Discussion

This consensus provides fresh insights into the use of ECCO_2_R for mitigating VILI in patients with ARDS. The group agreed that lung protective ventilation and ultra-protective lung ventilation should have distinct definitions, with ultra-protective lung ventilation primarily defined by a V_T_ 4–6 mL/kg PBW with a ΔP ≤ 14–15 cmH_2_O. ECCO_2_R may have a significant role in patients with ARDS by controlling hypercapnia and respiratory acidosis induced by these low V_T_ levels. While this provides a broad framework to help guide implementation of a protective or ultra-protective lung ventilation strategy supported by ECCO_2_R, it should be noted that the use of ECCO_2_R outside of randomized clinical trials is not recommended in the latest ESICM guidelines [29]. To this end, the group provided recommendations to guide the development of a trial to help overcome the uncertainties around the use of ECCO_2_R.

The previous ECCO_2_R Roundtable Meeting was held in July 2019. Since then, further insights into lung protective and ultra-protective lung ventilation and the use of ECCO_2_R have emerged. Firstly, the REST trial, the first large-scale randomized controlled trial of patients receiving MV facilitated by ECCO_2_R, was halted due to futility [26]. No significant benefit of ECCO_2_R on 90-day mortality vs. standard care was observed (41.5% vs. 39.5%, respectively; *p* = 0.68) [26]. In addition, serious adverse events were reported more commonly in the ECCO_2_R group, the majority related to bleeding complications, including intracranial hemorrhage. Bleeding complications associated with ECCO_2_R were indeed identified by our panel as one of the major endpoints to evaluate in a future trial of ECCO_2_R in ARDS. They also suggested unfractionated heparin should remain the first-line anticoagulant for ECCO_2_R, although new drugs with more favorable efficacy/safety profiles are currently under development and evaluation [31]. The REST trial had other major limitations. At randomization, ARDS was present in only 59% of the patients, ΔP was < 15 cm H_2_O in 50% of patients and, despite marked hypoxemia (median PaO_2_:FiO_2_, 118 mmHg), the median positive end-expiratory pressure (10 cm H_2_O) was lower than in other ARDS trials with similar patients and only 11% of the patients had been proned. On Day 2 post-randomization, the decreases in V_T_ (6.3–4.5 mL/kg) and in ΔP (15–12 cm H_2_O) from baseline were modest, while increases in the RR (from 24 to 27 BPM) and in PaCO_2_ (from 54 to 61 mmHg) were observed. These data suggest that the device used in this study may have provided insufficient CO_2_ removal to reach ultra-protective ventilation while controlling respiratory acidosis. Furthermore, as noted by the trial authors, most of the trial’s study sites were naive to the intervention, and inexperience may have negatively affected outcomes [26]. Interestingly, a secondary analysis of the REST trial has suggested a benefit of low V_T_ ventilation facilitated by ECCO_2_R on 90-day survival in a subset of the patients who had a high VR (> 3) and in patients with PaO_2_:FiO_2_ 110 mmHg or higher [28].

Secondly, the VT4COVID study conducted across 10 ICUs in France compared LTV (6 mL/kg PBW) or ULTV (4 mL/kg PBW) during the COVID-19 pandemic. There was no significant difference in the primary composite outcome of all-cause mortality at Day 90 and ventilator-free days at Day 60. Forty-six (44%) of 105 patients in the ULTV group and 43 (39%) of 109 in the LTV group died by Day 90 (absolute difference 4% [− 9 to 18]; p = 0.52) [30]. Severe respiratory acidosis in the first 28 days was higher in the ULTV group than in the LTV group (33% vs. 13%; absolute difference 20% [95% confidence interval 9–31]; p = 0.0004), suggesting the potential need for ECCO_2_R to facilitate ultra-protective lung ventilation. A major limitation of this trial is that the median ΔP was only 11 cm H_2_O at inclusion (with a modest reduction in ΔP of only 2 mm H_2_O in the ULTV group), with the benefit of further lowering V_T_ being very unlikely to outweigh the possible risks of heavy sedation, neuromuscular blockade, and diaphragm deconditioning. In a further development, the authors of the VT4COVID trial have since indicated that they are analyzing their trial database to identify a threshold of ΔP above which ULTV would be beneficial [30]. Another limitation of the VT4COVID trial is that the possible benefit of the reduction in TV in the ULTV group may have been masked by the increase of respiratory rates, with such increases in respiratory rates potentially a consequence of respiratory acidosis and hypercapnia in this population. Although not necessarily supporting the concept of ULTV, these observations may suggest that its application without sufficient extracorporeal reduction of CO_2_ load may limit its beneficial effects for patients.

Thirdly, the ESICM guidelines on ARDS recommended against the use of ECCO_2_R for the treatment of ARDS outside of randomized controlled trials [29]. This is based on a meta-analysis of the primary analysis of the REST trial and the smaller Xtravent trial, which suggests the use of ECCO_2_R did not reduce mortality as well as the side effects experienced in the REST trial in patients receiving ECCO_2_R. However, the ESICM experts do acknowledge the need for further research to clarify the current uncertainty around ECCO_2_R; specifically, understanding device-specific safety and efficacy profiles, the identification of long-term multidimensional outcomes, and defining a population of patients with ARDS who may respond to ECCO_2_R [29]. Indeed, during our meeting, there was a recurring discussion on the importance of a patient-centric approach to ECCO_2_R, highlighting that the parameters necessary for initiation and discontinuation of protective ventilation and/or ECCO_2_R must be adapted to the patient’s disease severity, comorbidities, and ventilatory parameters associated with lung injury. Specifically, the participants emphasized that parameters reflecting alterations in lung mechanics, such as an increase in ΔP, might serve as better inclusion criteria for ECCO_2_R in patients with acute hypoxemic respiratory failure than the degree of hypoxemia. The group also believed that a minimum CO_2_ removal rate of 80 mL/min delivered by continuous renal support machines was required for ECCO_2_R to be effective, with a short, double-lumen catheter inserted into the right internal jugular vein as the preferred vascular access. Furthermore, we note the type of device use to deliver ECCO_2_R may have some import. That is, at the flow rates typically used for ECCO_2_R (~ 300 to ~ 1500 mL/min), centrifugal devices (used in the REST trial) [26] have an associated risk of hemolysis and destruction of platelets [32] that may not occur with peristaltic pumps. Our recommendations provided here on the use of ECCO_2_R and on clinical trial design should aid the development of a trial that could help answer the questions posed by the ESICM guidelines on ARDS.

This work does have some limitations. Firstly, the findings relate to the experiences of a relatively small number of physicians from centers across Europe and do not replace the need for a randomized controlled trial to determine the optimal use of ECCO_2_R to facilitate LTV strategies. Secondly, the group focused on the use of ECCO_2_R to facilitate ventilation in patients with ARDS—these experiences may not translate to other rarer indications. Thirdly, we did not include discussions related to the amount of CO_2_ removal required depending on ideal/predicted body weight or other external factors that could influence a patient’s CO_2_ production. The amount of CO_2_ that is produced by the patient is dependent on multiple factors (such as muscle activity, inflammatory reactions and nutrition). As a result, it cannot be defined easily and there is no method available that can be used easily at the bedside to determine this production rate reliably. This is another reason why more physiological and interventional studies are needed to explore the ability of the ECCO_2_R device to control hypercapnia while providing ultra-protective lung ventilation. Although the authors took every opportunity to ensure all relevant major articles were cited when constructing surveys, a comprehensive systematic literature analysis was considered out of scope of this project. Readers are reminded that the discussions outlined here are the authors’ personal experiences and are not a replacement for formal guidelines. Practicing clinicians should continue to prioritize their patients’ individual needs and consult guidelines.

## Conclusions

The authors consider that ECCO_2_R may facilitate UPLV with lower volume and pressures by the ventilator while controlling respiratory acidosis. ECCO_2_R may be delivered using blood flows currently delivered by continuous renal support machines, providing that a minimum CO_2_ removal rate of 80 mL/min can be obtained. Since recent ESICM guidelines on ARDS recommended against the use of ECCO_2_R for the treatment of ARDS outside of randomized controlled trials, a new trial of ECCO_2_R is now urgently needed (with ΔP of ≥ 14–15 cmH_2_O suggested as the primary inclusion criterion).

### Supplementary Information

Additional file 1.

## Data Availability

All data generated or analyzed during this study are included in this published article [and its supplementary information files].

## Abbreviations

∆P: Driving pressure
ARDS: Acute respiratory distress syndrome
BPM: Breaths per minute
ECCO_2_R: Extracorporeal CO_2_ removal
ESICM: European Society of Intensive Care Medicine
FiO_2_: Fraction of inspired oxygen
ICU: Intensive care unit
LTV: Low V_T_ ventilation
MV: Mechanical ventilation
PaCO_2_: Partial pressure of CO_2_
PaO_2_: Partial pressure of arterial oxygen
P_Plat_: Plateau pressure
RR: Respiratory rate
ULTV: Ultra-low V_T_ ventilation
UPLV: Ultra-protective lung ventilation
VILI: Ventilator-induced lung injury
VR: Ventilatory ratio
V_T_: Tidal volume
