# Supplementary material for: Expert perspectives on ECCO2R for acute hypoxemic respiratory failure: consensus of a 2022 European roundtable meeting
Source: Ann Intensive Care. 2024 Aug 22;14:132. doi: 10.1186/s13613-024-01353-8 (PMC11341504; doi:10.1186/s13613-024-01353-8)
Supplement: Supplementary file 1 — Additional file 1. [file 13613_2024_1353_MOESM1_ESM.docx]

**Supplementary materials**

1. **Pre-meeting survey questions**

Participants were required to select a multiple-choice response and/or use the free text to write in their responses where applicable.

***i. Professional background***

1. a) What type of unit do you work at? Please check all that apply.

- Medical intensive care unit (ICU)
- Surgical ICU
- Combined medical and surgical ICU
- Cardiac surgical ICU
- Respiratory acute care
- Other (please specify: ____________)

b) How many beds are there in your ICU/unit?

- 1–10
- 11–20
- 21–30
- 31–40
- 41–50
- 51–60
- 60+ (please specify: ____________)

c) On average, how many patients are admitted to your ICU/unit per year?

- 1–200
- 201–400
- 401–600
- 601–800
- 801–1,000
- 1,001–1,250
- 1,251–1,500
- 1,501–1,750
- 1,751–2,000
- 2,000+ (please specify: ____________)

2. How many patients with acute exacerbation of chronic obstructive pulmonary disease (aeCOPD) are admitted to your ICU/unit per year?

- 0
- 1–10
- 11–20
- 21–30
- 31–40
- 41–50
- 51–60
- 60+ (please specify: ____________)

a) What proportion of these patients are intubated before admission to your ICU/unit?

- 0%
- 1–10%
- 11–20%
- 21–30%
- 31–40%
- 41–50%
- 51–60%
- 61–70%
- 71–80%
- 81–90%
- 91–99%
- 100%

***ii. Mechanical ventilation***

3. Does your center have an established protocol for when and/or how to implement mechanical ventilation in patients with hypoxemic acute respiratory failure?

- Yes
- No

If you answered “Yes”:

a) Is there a target tidal volume (V_T_)? Please specify values if so: __________________________________________________________________________

b) Is there a target respiratory rate? Please specify values if so: __________________________________________________________________________

c) Is the target positive end-expiratory pressure (PEEP) in alignment with the ARDSnet protocol?

- Yes
- No

d) Does your center’s protocol include instructions for weaning patients from ventilation?

- Yes
- No

e) Have there been any significant changes to your center’s protocol as a result of the COVID-19 pandemic?

- Yes
- No

f) Does your center’s protocol include a lung recruitment maneuver?

- Yes
- No

g) Does your center’s protocol suggest use of an electrical impedance tomography (EIT) device to optimize ventilation parameters?

- Yes
- No

4. Which respiratory criteria do you consider most important to monitor when trying to minimize or avoid ventilator-induced lung injury (VILI)?

Please rank in order of importance, with “1” being the most important.

_____ V_T_

_____ Driving pressure

_____ plateau pressure (P_plat_)

_____ PEEP

_____ Mechanical power

_____ Static/dynamic compliance

_____ Other (please state: ____________)

5. Are lung-protective ventilation (LPV) (V_T_ ~6 mL/kg) or ultra-protective lung ventilation (UPLV) (V_T_ ~4 mL/kg) used in your ICU/unit?

- No, neither are used
- Yes, LPV is used
- Yes, UPLV is used
- Yes, both are used

***iii. Extracorporeal CO_2_ removal (ECCO_2_R) therapy experience and clinical practice***

6. On average, how many patients are treated with ECCO_2_R therapy in your ICU/unit per year?

- 1–2
- 3–4
- 5–6
- 7–8
- 9–10
- 11–12
- 13–14
- 15–16
- 17–18
- 19–20
- 20+ (please state: ____________)

7. Please rank the five primary clinical indications for ECCO_2_R therapy in your ICU/unit, with “1” being the most common indication (e.g. acute respiratory distress syndrome [ARDS], aeCOPD, severe asthma).

1_________

2_________

3_________

4________

5________

***iv. ECCO_2_R therapy in ARDS***

8. On average, how many patients with mild-to-moderate ARDS (as defined by the 2012 Berlin Definition) are admitted to your ICU/unit per year?

- 1–20
- 21–40
- 41–60
- 61–80
- 81–100
- 101–120
- 121–140
- 141–160
- 161–180
- 181–200
- 200+ (please state: ____________)

9. During the Expert User Group Meeting in 2019, participants considered the primary treatment goal of ECCO_2_R therapy for patients with ARDS to be “application of UPLV via managing CO_2_ levels.” Given the potential impact of the COVID-19 pandemic on clinical practice and recent published literature, do you agree or disagree that this is still the primary treatment goal of ECCO_2_R therapy in patients with ARDS?

- Agree
- Disagree

a) If “Disagree,” what do you consider to be the primary treatment goal of implementing ECCO_2_R therapy in patients with ARDS? (please state: ____________)

***v. Initiation of ECCO_2_R therapy in patients with ARDS***

10. Which of the following criteria most influence your decision whether to initiate ECCO_2_R therapy in sedated and ventilated patients with mild-to-moderate ARDS?

Please rank in order of importance, with “1” being the most important.

_____ Driving pressure

_____ P_plat_

_____ partial pressure of CO_2_ (PaCO_2_)

_____ pH

_____ Plan to reduce V_T_ to <6 mL/predicted body weight (PBW)

_____ Respiratory rate

_____ partial pressure of arterial oxygen (PaO_2_)/ fraction of inspired oxygen (FiO_2_)

_____ PEEP

_____ Mechanical power

_____ Dead space fraction

_____ Static/dynamic compliance

***vi. Discontinuation of ECCO_2_R therapy in patients with ARDS***

11. Which of the following criteria do you believe are important to consider when discontinuing ECCO_2_R therapy as part of a general de-escalation of respiratory support in sedated and ventilated patients with mild-to-moderate ARDS?

Please rank in order of importance, with “1” being the most important.

_____ Driving pressure

_____ P_plat_

_____ Respiratory rate

_____ pH

_____ V_T_

_____ PaCO_2_

_____ Dead space fraction

_____ Static/dynamic compliance

***vii. ECCO_2_R therapy: practical considerations***

12. Which of the following criteria are most important to monitor and/or adapt during use of ECCO_2_R therapy in sedated and ventilated patients?

Please rank in order of importance, with “1” being the most important.

_____ Driving pressure

_____ P_plat_

_____ PaCO_2_

_____ pH

_____ V_T_

_____ Respiratory rate

_____ PaO_2_/FiO_2_

_____ PEEP

_____ Mechanical power

_____ Dead space fraction

_____ Airway pressure

13. Which method of anticoagulation do you primarily use during ECCO_2_R therapy in sedated and ventilated patients? Please consider drug, dosage, etc.

_________________________________________________________________________________

14. Do you use neuromuscular blockade during ECCO_2_R therapy in sedated and ventilated patients?

- Yes
- No

15. Which form of vascular access do you primarily use during ECCO_2_R therapy (please consider access point, cannula type, length, size, etc.):

a) in sedated and ventilated patients? _________________________________________________________________________________

b) in non-sedated patients? _________________________________________________________________________________

16. What blood flow rate range do you primarily use during ECCO_2_R therapy:

a) in sedated and ventilated patients? _________________________________________________________________________________

b) in non-sedated patients? _________________________________________________________________________________

17. What do you perceive to be the main challenge(s) to the implementation of ECCO_2_R therapy in your ICU/unit?

Please rank in order of importance, with “1” being the most important.

_____ Therapeutic indications for ECCO_2_R therapy not well understood

_____ Insufficient product supply

_____ Perceived risk of complications/
adverse events

_____ Unclear/uncertain of clinical value of ECCO_2_R therapy

_____ Costs and resources associated with the use of ECCO_2_R therapy

_____ Staffing shortages

_____ Lack of training

_____ Other (please state: ____________)

18. What do you consider to be the acceptable minimum CO_2_ removal rate for an ECCO_2_R device?

- 30–50 mL/min
- 51–80 mL/min
- 81–100 mL/min
- 101–120 mL/min
- >120 mL/min

19. Is the Baxter **PrismaLung+** gas exchanger used to treat patients with ECCO_2_R therapy in your ICU/unit?

- Yes
- No

a) If you answered “Yes,” how many patients on average are treated with **PrismaLung+** in your ICU/unit per year? __________________________________________________________

b) If you answered “No,” which other devices do you use to perform ECCO_2_R therapy? Please list all product names: ________________________________________________________________

***viii. ECCO_2_R therapy in combination with renal support***

20. Is ECCO_2_R therapy ever used in combination with continuous renal replacement therapy (CRRT) for patients in your ICU/unit?

- Yes
- No

21. What proportion of patients receiving ECCO_2_R therapy in your ICU/unit simultaneously receive CRRT therapy?

- 0–10%
- 11–20%
- 21–30%
- 31–40%
- 41–50%
- 51–60%
- 61–70%
- 71–80%
- 81–90%
- 91–100%

22. What do you perceive to be the clinical rationale for using ECCO_2_R therapy and CRRT simultaneously?

_________________________________________________________________________________

1. **ECCO_2_R Expert User Group Meeting survey questions**

Participants answered questions during the meeting using an interactive voting system. Participants were required to select a multiple-choice response and/or use the free text to write in their responses where applicable.

***i. Respiratory criteria***

1. Which respiratory criteria do you consider most important to monitor when trying to minimize or avoid VILI?
Please rank in order of importance, with “1” being the most important.

_____ V_T_

_____ Driving pressure

_____ P_plat_

_____ PEEP

_____ Mechanical power

_____ Static/dynamic compliance

_____ Respiratory rate

***ii. Defining LPV***

2) Which parameters should be used to define LPV?
Please provide the target value or range for each parameter or leave blank if you do not think it should be included. If “other,” please specify both parameter and target value/range.

_____ V_T_

_____ Driving pressure

_____ P_plat_

_____ PEEP

_____ Respiratory rate

_____ Other

***iii. Defining UPLV***

3) Which parameters should be used to define UPLV?
Please provide the target value or range for each parameter or leave blank if you do not think it should be included. If “other,” please specify both parameter and target value/range.

_____ V_T_

_____ Driving pressure

_____ P_plat_

_____ PEEP

_____ Respiratory rate

_____ Other

***iv. ECCO_2_R therapy in ARDS***

4) Based on earlier discussions on the definitions of LPV and UPLV, do you agree or disagree that the “application of ultra-protective lung ventilation (UPLV) via managing CO_2_ levels” is still the primary treatment goal of ECCO_2_R therapy in patients with ARDS?

- Agree
- Disagree

***v. Initiation criteria in ARDS***

5) What are the relevant criteria for initiation of ECCO_2_R therapy in sedated and ventilated patients with mild-to-moderate ARDS?

Please rank in order of importance, with “1” being the most important.

Driving pressure ____

____ P_plat_

____ PaCO_2_

____ pH

____ Plan to reduce V_T_ to <6 mL/PBW

____ Respiratory rate

____ PaO_2_/FiO_2_

____ PEEP

____ Mechanical power

____ Dead space fraction

____ Static/dynamic compliance

6) What are the relevant criteria for initiation of ECCO_2_R therapy in sedated and ventilated patients with mild-to-moderate ARDS?

Please provide the threshold value for each criterion or leave blank if you do not think it should be prioritized.

__________ Driving pressure

__________ P_plat_

__________ PaCO_2_

__________ pH

__________ Plan to reduce V_T_ to <6 mL/PBW (yes/no)

__________ Respiratory rate

__________ PaO_2_/FiO_2_

__________ PEEP

__________ Mechanical power

__________ Dead space fraction

__________ Static/dynamic compliance

***vi. Discontinuation criteria in ARDS***

7) What are the relevant criteria for discontinuation of ECCO_2_R therapy as part of a general
de-escalation of respiratory support in sedated and ventilated patients with mild-to-moderate ARDS?

Please rank in order of importance, with “1” being the most important.

____ Driving pressure

____ P_plat_

____ Respiratory rate

____ pH

____ V_T_

____ PaCO_2_

____ Dead space fraction

____ Static/dynamic compliance

8) What are the relevant criteria for discontinuation of ECCO_2_R therapy as part of a general
de-escalation of respiratory support in sedated and ventilated patients with mild-to-moderate ARDS?

Please provide the target value or range for each criterion or leave blank if you do not think it should be prioritized.

__________ Driving pressure

__________ P_plat_

__________ Respiratory rate

__________ pH

__________ V_T_

__________ PaCO_2_

__________ Dead space fraction

__________ Static/dynamic compliance

9) What do you think should be the average duration of an ECCO_2_R treatment in sedated and ventilated patients with mild-to-moderate ARDS?

_________________________________________________________________________________

***vii. Clinical experience with PrismaLung+***10) What is your clinical experience of using the PrismaLung+ device?

_________________________________________________________________________________

***viii. Anticoagulation***11) Please describe your recommended protocol for using unfractionated heparin anticoagulation for ECCO_2_R therapy in sedated and ventilated patients.

Please provide specific values/ranges or leave blank if you do not think it should be included.

 Dose _______ U/kg bolus

Dose ______ U/kg/hr continuous infusion

Target activated partial thromboplastin time (aPTT) ­­­­­­­­­­­­­­­­­______

Target anti-Xa ­­­­­­­­­­­______

Target thromboelastography (TEG) ______

Target antithrombin III (AT III) ______

***ix. Neuromuscular blockade***12) Do you routinely use neuromuscular blockade during ECCO_2_R therapy in sedated and ventilated patients with ARDS?

- Yes
- No

13) What would be your indication for use of a neuromuscular blockade during ECCO_2_R therapy in sedated and ventilated patients with ARDS?

_________________________________________________________________________________

***x. Vascular access and blood flow***14) What would be your preferred minimum blood flow rate when using ECCO_2_R therapy?

- >150 mL/min
- >200 mL/min
- >250 mL/min
- >300 mL/min
- >350 mL/min
- >400 mL/min
- >450 mL/min
- >500 mL/min
- >550 mL/min

15) To achieve this blood flow rate, what would be your preferred access point?

Please rank in order of importance, with “1” being the most important.

___ Right internal jugular vein

___ Left internal jugular vein

___ Femoral vein

___ Subclavian vein

16) To achieve this blood flow rate, what would be your preferred cannula type?
__________________________________________________________________ (Please describe)

17) To achieve this blood flow rate, what would be your preferred cannula size?

- 12 Fr
- 13 Fr
- 14 Fr
- 15 Fr
- 16 Fr

18) To achieve this blood flow rate, what would be your preferred cannula length?

- 16–17 cm
- 18–19 cm
- 20–21 cm
- 22–23 cm
- 24–25 cm
- 25+ cm

19) What do you consider to be a valid vascular assessment?

 ________________________________________________________________________________20) What are the challenges you’ve experienced with initiation and maintenance of vascular access to achieve target blood flow?

_________________________________________________________________________________

***xi. Minimum acceptable CO_2_ removal rate***21) What do you consider to be the acceptable minimum CO_2_ removal rate for an ECCO_2_R device?

- 50 mL/min
- 55 mL/min
- 60 mL/min
- 65 mL/min
- 70 mL/min
- 75 mL/min
- 80 mL/min
- 85 mL/min
- 90 mL/min
- 95 mL/min
- 100 mL/min
- 105 mL/min
- 110 mL/min
- 115 mL/min
- 120 mL/min
- >120 mL/min

22) What would be your expectations for a novel ECCO_2_R device?

_________________________________________________________________________________

23) What is your clinical experience of using the PrismaLung+ device?

_________________________________________________________________________________

1. **Post-meeting survey questions (prepared November 2022)**

Participants were required to select a multiple-choice response and/or use the free text to write in their responses where applicable.

***i. Framework for protective ventilation in ARDS***1. When implementing protective ventilation to minimize or avoid VILI for a patient with mild‑to‑moderate ARDS, what would be the acceptable threshold value for the following parameters? Please select one option for each parameter.

a) Minimum V_T_ (mL/kg ideal body weight [IBW])

- 3
- 4
- 5
- 6
- 7
- 8
- >8

b) Maximum respiratory rate (BPM)

- ≤10
- 11–15
- 16–20
- 21–25
- 26–30
- >30

c) Maximum driving pressure (cmH_2_O)

- <13
- 13
- 14
- 15
- 16
- 17
- 18
- 19
- 20

d) Maximum P_plat_ (cmH_2_O)

- <25
- 25–26
- 27–28
- 29–30
- >30

e) To achieve this protective ventilation strategy, would you require ECCO_2_R therapy?

- Yes
- No

***ii. Framework for UPLV in ARDS***2. Do you believe that LPV and UPLV should have distinct definitions?

- Yes
- No

Please explain your rationale. If “Yes,” how would you define the differences between them? Please consider possible differences in threshold values for respiratory parameters.

_____________________________________________________________________________

***iii. Initiation of ECCO_2_R therapy in ARDS***3. Please specify the threshold value for *initiation* of ECCO_2_R therapy in a sedated and ventilated patient with mild-to-moderate ARDS. Please select one option for each parameter.

a) Driving pressure (cmH_2_O)

- >14
- >15
- >16
- >17
- >18
- >19
- >20

b) Respiratory rate (BPM)

- >15
- >16
- >17
- >18
- >19
- >20
- >21
- >22
- >23
- >24
- >25
- >26
- >27
- >28
- >29
- >30

c) PaCO_2_ (mmHg)

- >60
- >65
- >70
- >75
- >80

d) pH

- <7.2
- <7.25

***iv. Discontinuation of ECCO_2_R therapy in ARDS***4. Please specify the threshold value for *discontinuation* of ECCO_2_R therapy in a sedated and ventilated patient with mild-to-moderate ARDS. Please select one option for each parameter.

a) Driving pressure (cmH_2_O)

- <12
- <13
- <14
- <15

b) P_plat_ (cmH_2_O)

- <22–23
- <24–25
- <26–27
- <28–29
- <30

c) Respiratory rate (BPM)

- <20
- <21
- <22
- <23
- <24
- <25

d) pH

- >7.25
- >7.3
- >7.35

***v. Anticoagulation***Please describe your recommended protocol for using unfractionated heparin anticoagulation for ECCO_2_R therapy in sedated and ventilated patients. Please select one option for each parameter. If you do not use unfractionated heparin as an anticoagulant for ECCO_2_R therapy, please skip this question.

a) Bolus dose (units/kg)

- I do not use a bolus dose
- 40–50
- 51–60
- 61–70
- 71–80

b) Infusion dose (units/kg/hr)

- I do not use an infusion
- 6–8
- 9–11
- 12–14
- 15–17

c) Target aPTT level (ratio vs. reference range)

- I do not routinely use aPTT monitoring
- <1.5
- 1.5–2.0
- >2.0

d) Target anti-Xa level (units/mL)

- I do not routinely use
  anti-Xa monitoring
- 0.2–0.4
- 0.3–0.5

***vi. Neuromuscular blockade***6. Do you routinely use neuromuscular blockade for sedated patients with ventilator asynchrony receiving ECCO_2_R therapy?

- Yes
- No

***vii. Prone positioning***7. Do you routinely use prone positioning for sedated and ventilated patients with mild-to-moderate ARDS receiving ECCO_2_R therapy?

- Yes
- No

***viii. Blood flow rate***8. What is the *minimum* blood flow rate required for effective use of ECCO_2_R therapy? Please select one option.

- 150–250 mL/min
- 251–350 mL/min
- 351–450 mL/min
- >450 mL/min

***ix. Vascular access point***9. Please rank your preferred vascular access point for ECCO_2_R therapy, with “1” being your first choice (assuming that all options are available in a patient).

___ Right internal jugular vein

___ Femoral vein

___ Left internal jugular vein

___ Subclavian vein

***x. Catheter type***10. What is your preferred catheter type for ECCO_2_R therapy?

- Double-lumen catheter
- Two single-lumen catheters

***xi. Catheter size and length***11. What would be your preferred catheter size and length for ECCO_2_R therapy? Please select one option for size and one option for length for each access point.

a) Right internal jugular vein

Size (Fr):

- 13
- 14

Length (cm):

- 16–17
- 18–19
- 20–21
- 22–23
- 24–25

b) Femoral vein

Size (Fr):

- 13
- 14

Length (cm):

- 16–17
- 18–19
- 20–21
- 22–23
- 24–25

c) Left internal jugular vein

Size (Fr):

- - - 13
    - 14

Length (cm):

- - - 16–17
    - 18–19
    - 20–21
    - 22–23
    - 24–25

d) Subclavian vein

Size (Fr):

- - - 13
    - 14

Length (cm):

- - - 16–17
    - 18–19
    - 20–21
    - 22–23
    - 24–25

***xii. Vascular assessment***12. Is ultrasound/sonography your preferred form of vascular assessment during ECCO_2_R therapy?

- - Yes
  - No, I prefer to use ________________________

***xiii. Future research in ARDS***13. Do you believe a new randomized trial of ECCO_2_R therapy is needed in patients with ARDS? If “No,” please skip question 14.

- - Yes
  - No

***xiv. Methodology for future research in ARDS***14. If you were to design a new randomized controlled trial of ECCO_2_R therapy in patients with ARDS, what would be your suggested methodology?

a) Definition of ARDS for inclusion:

PaO_2_/FiO_2_ >_______ mmHg

PaO_2_/FiO_2_ ≤_______ mmHg

PEEP or continuous positive airway pressure (CPAP) ≥ ________ cmH_2_O

b) Driving pressure for inclusion: ☐ ≥_____ mmHg ☐ No minimum driving pressure

c) Describe any other suggested inclusion criteria: _________________________________________________________________________________

d) Describe any suggested exclusion criteria: _________________________________________________________________________________

e) Describe your suggested protocol: _________________________________________________________________________________

i. Would your protocol include use of prone positioning? If so, in which patients? _____________________________________________________________________

f) Describe your suggested primary endpoint: _________________________________________________________________________________

g) Describe any other suggested endpoints: _________________________________________________________________________________

h) Describe any suggested safety endpoints: _________________________________________________________________________________

i) Additional comments: _________________________________________________________________________________

1. **Post-meeting survey questions (prepared August 2022)**

Participants were required to select a multiple-choice response.

***i. Definition for UPLV***1. Based on the latest evidence (secondary analysis of the REST trial, guidelines for ARDS treatment, VT4COVID trial) that has been published since the User Group Meeting in October 2022, do you still define UPLV as having a minimum V_T_ <6 mL/kg PBW?

- - Yes
  - No

If you selected NO, what V_T_ threshold would you use to define UPLV?

- - ≤5 mL/kg PBW
  - ≤4 mL/kg PBW
  - ≤3 mL/kg PBW
  - ≤2 mL/kg PBW

***ii. Use of ECCO_2_R in patients with UPLV***2. Based on the latest evidence (secondary analysis of the REST trial, guidelines for ARDS treatment, VT4COVID trial) that has been published since the User Group Meeting in October 2022, do you believe that ECCO_2_R therapy will be needed in most patients in order to implement UPLV?

- Yes
- No

***iii. Patient populations for ECCO_2_R and UPLV***3. Based on the latest evidence (secondary analysis of the REST trial, guidelines for ARDS treatment, VT4COVID trial) that has been published since the User Group Meeting in October 2022, which patient population would you select for applying a strategy of UPLV permitted by ECCO_2_R therapy?

a) Minimum driving pressure (ΔP)?

- Yes
- No

If you selected “yes,” then what threshold would you use?

- 12–13 mmHg
- 14–15 mmHg
- 16–17 mmHg
- >17 mmHg

b) Minimum PaO_2_:FiO_2_ >100 mmHg?

- Yes
- No

c) Minimum respiratory ratio >2.5–3?

- Yes
- No
